# Parent Satisfaction With Outpatient Telemedicine Services During the COVID-19 Pandemic: A Repeated Cross-Sectional Study

**DOI:** 10.3389/fped.2022.908337

**Published:** 2022-08-25

**Authors:** Erin Jones, Jaime Kurman, Elisa Delia, Jennifer Crockett, Rachel Peterson, Jasmin Thames, Cynthia Salorio, Luther Kalb, Lisa Jacobson, Jacqueline Stone, T. Andrew Zabel

**Affiliations:** ^1^Department of Neuropsychology, Kennedy Krieger Institute, Baltimore, MD, United States; ^2^Office of Patient Experience and Community Engagement, Kennedy Krieger Institute, Baltimore, MD, United States; ^3^Administration, Kennedy Krieger Institute, Baltimore, MD, United States; ^4^Johns Hopkins University School of Medicine, Johns Hopkins University, Baltimore, MD, United States; ^5^Johns Hopkins Bloomberg School of Public Health, Johns Hopkins University, Baltimore, MD, United States; ^6^Center for Autism and Related Disorders, Kennedy Krieger Institute, Baltimore, MD, United States

**Keywords:** telemedicine, telehealth, pediatrics, quality improvement, COVID-19 pandemic, patient experience, satisfaction

## Abstract

Prior to the COVID-19 pandemic, the development of hospital-based telemedicine services had been slow and circumscribed in scope due to insurance and licensure restrictions. As these restrictions were eased during the COVID-19 pandemic to facilitate ongoing patient care, the public health emergency facilitated a rapid expansion and utilization of telemedicine services across the ambulatory service sector.

**Objectives:**

The current quality improvement (QI) study utilized this unprecedented opportunity to evaluate the use of telemedicine services across a variety of clinical disciplines and patient groups.

**Methods:**

Caregivers of patients (ages 0–21) who received care through an outpatient specialty center provided experience ratings of telemedicine services delivered during the initial pandemic months (March–June 2020; *N* = 1311) or during the national “winter surge” in late 2020 (November 2020–February 2021; *N* = 1395). Questionnaires were distributed electronically following the clinical visits, and ANCOVA was employed (with patient age as the covariate) to determine if caregiver responses differed based on patient demographic characteristics.

**Results:**

Ratings of patient satisfaction with services were very strong at both time points; greater variability in scores was noted when caregivers were asked if they would use telemedicine services again. At both time points, younger patient age (i.e., age 0–5) was associated with decreased caregiver willingness to use telemedicine services in the future. Smaller effects were seen for certain “hands on” therapies (occupational, physical, and speech) during the initial months of the pandemic and for proximity to the hospital during the “winter surge.”

**Conclusions:**

These data suggest a very positive overall caregiver response to telemedicine-based services during the COVID-19 pandemic. Several areas of potential improvement/innovation were identified, including the delivery of telemedicine therapies (e.g., occupational, physical, and speech) services to young patients (i.e., aged 0–5).

## Introduction

The onset of the COVID-19 pandemic and the resulting federal Public Health Emergency (PHE) declaration led to several notable changes in pediatric medicine, including an unprecedented and rapid shift from in-person to telemedicine services to continue to facilitate patient care while maintaining a physical distance. Telemedicine includes two-way, real-time interactive communication using audio and video equipment between the patient and practitioner at a distant site (1). The PHE led to the passage of PL 116-123, with a Section 1135 waiver, allowing for the temporary waiver of certain Medicare requirements. This established the path for Health and Human Services (HHS) to introduce several flexibilities that permitted telemedicine accessibility, e.g., expansion of eligible practitioners and covered services, removal of restrictions for patient and practitioner location, easing of technology requirements by the Health Insurance Portability and Accountability Act (HIPAA), permission to practice across state lines, and the ability to bill as if the session was conducted in person (2). These changes in restrictions helped facilitate an exponential increase in the provision of telemedicine services (3, 4). With these changes, medical centers were quickly faced with the need to establish the infrastructure necessary to deliver, support, and evaluate their telemedicine services (5).

Telemedicine can be advantageous for patients and caregivers. First, telemedicine may reduce the burden on patients and their families by reducing time away from school, missed time at work, and/or travel time associated with in-person services (6–10). The time-saving convenience of telemedicine can extend to registration, triage, and wait times for patients as those components are often taken care of in advance of a telemedicine appointment (6).

While general satisfaction with telemedicine-based care has typically been high, recent studies have found a discrepancy between expressed satisfaction and willingness to use telemedicine-based services again (9, 11, 12). For instance, Leung et al. (11) found that 96% of neurosurgical patients rated their telemedicine visit as “excellent” or “good,” but only 83% of patients indicated they would choose a video visit over an in-person visit. Similarly, Schmidtberg et al. (12) documented very high satisfaction ratings (95.6%), but found that fewer (87%) patients would consider another telemedicine visit in the future. These findings raise questions concerning the small, but noteworthy, a cohort of patients and caregivers who are more hesitant about using telemedicine beyond their initial experience.

Hesitancy concerning the reuse of telemedicine may derive in part from care-related variables. Tomines (13) found that telemedicine's effectiveness varied widely based upon the pediatric specialty, care delivery setting, and patient preference. Tenforde et al. (9) qualitatively identified several parents- and family members-reported limitations of rehabilitation services delivered via telemedicine, such as the inability of the therapist to receive tactile motor/muscle feedback during a telemedicine visit. In addition, younger children may have difficulty engaging with a provider or staying focused during a telemedicine appointment (9) and require assistance from a parent or caregiver throughout the telemedicine appointment (14, 15). This parent/caregiver participation can result in additional burdens for caregivers who may be simultaneously working or caring for other children (16).

This quality improvement (QI) project was designed to assess parent and caregiver satisfaction with telemedicine appointments and related services during the COVID-19 pandemic. The aims of this study were to: (1) examine parent satisfaction with telemedicine care at two-time points during the COVID-19 pandemic; and (2) identify parent-, child-, and service-related variables associated with parent satisfaction with telemedicine services. We hypothesized that parents/caregivers would be less interested in using telemedicine again if their children were younger (e.g., ages 0–5), were seen for “hands on” therapy services (i.e., occupational therapy and physical therapy), and/or lived near the hospital. While several studies have looked at the provision of telemedicine services in specific disciplines, this study aimed to evaluate the use of telemedicine services across multiple disciplines.

## Methods

This quality improvement study received acknowledgment from the hospital's overseeing institutional review board (IRB), as well as oversight by the internal Office of Human Research Administration.

### Facility

This study took place in an interdisciplinary hospital specializing in treatment for children and young adults referred for medical, cognitive, and behavioral issues related to traumatic injuries or intellectual/developmental disabilities. The hospital had a pre-existing telebehavioral health program for military families that was established 4 years prior to the COVID-19 pandemic. Although this was a small program (100 patients, 21 telehealth providers, and 700 sessions), it provided the infrastructure necessary to rapidly shift to telemedicine-based services delivered in families' homes when the pandemic began. The hospital had telemedicine policies and procedures already in place, as well as established HIPAA-compliant video conferencing and electronic documentation programs. The experienced telebehavioral health providers were able to transition their efforts to expand the established systems throughout the hospital, such that by the end of March 2020, the hospital had provided over 4000 telehealth sessions.

### Participants

Caregivers responded to a brief experience questionnaire following their child's outpatient telemedicine appointment. A repeated cross-sectional design resulted in data at two-time points (Table 1). The utilization of two samples allowed for the examination of caregiver experience during the initial transition to telemedicine, as well as experience deeper into the pandemic when clinicians, parents/caregivers, and patients were potentially more familiarized with telemedicine-based services. Caregiver responses were included if they responded to both target questions, identified in the *Questionnaire* section that follows.

**Table 1:** Demographic composition of the Time 1 (Early Pandemic) and Time 2 (Winter Surge) groups.

	**Time 1**	**Time 2**
**Sex**, ***n*** **(%)**		
Male	879 (67.0)	847 (60.6)
Female	432 (33.0)	548 (39.1)
**Age groups**, ***n*** **(%)**		
0–5	264 (20.1)	318 (22.8)
6–10	437 (33.3)	459 (32.9)
11–15	386 (29.5)	408 (29.2)
16–21	224 (17.1)	210 (15.1)
**Race**, ***n*** **(%)**		
White	666 (50.8)	683 (49.0)
Black	377 (28.8)	361 (25.9)
Other	171 (13.1)	104 (7.5)
Multiracial	33 (2.5)	119 (8.5)
Unknown	64 (4.8)	127 (9.1)
**Insurance**, ***n*** **(%)**		
Commercial	804 (61.3)	857 (61.5)
Public	380 (29.0)	420 (30.2)
Military	127 (9.7)	116 (8.3)
**Service type**, ***n*** **(%)**		
BH: Community	407 (31.0)	622 (44.6)
BH: Developmental	153 (11.7)	240 (17.2)
Medicine	512 (39.1)	397 (28.5)
Therapy Services	239 (18.2)	136 (9.7)
**Proximity to hospital**, ***n*** **(%)**		
Close Proximity	197 (15.0)	163 (11.9)
Adjacent/Surrounding	643 (48.0)	715 (52.4)
Distal	471 (35.9)	487 (35.7)

*BH, Behavioral Health*.

### Time 1, Early Pandemic Sample

The *Time 1, Early Pandemic* sample was defined as the patients seen during the first several months of the COVID-19 pandemic (13 March 2020–13 May 2020). A single retrospective distribution of telemedicine experience questionnaires returned 2128 responses (24.9% completion rate) during the data collection window of June 1–June 30, 2020. Of these, 223 unfinished or duplicate responses were removed, leaving 1872 responses. To focus on caregiver reports, patient reports were removed which further reduced the sample to 1535 responses. The few (10) self-pay patients were removed as well. Finally, the sample was reduced to 1311 respondents after 214 surveys with missing or skipped responses were removed. Demographic information of the patients in the early pandemic sample is presented in Table 1.

### Time 2, Winter Surge Sample

The *Time 2, Winter Surge* sample was defined as the patients seen during a portion of the national “winter surge” of COVID-19 cases (20 November 2020–26 February 2021). Surveys were distributed on an ongoing basis during the sampling window of 8 December 2020 and 2 March 2021. Of the 3,188 questionnaires received (11.9% completion rate), 1131 unfinished or duplicate surveys were removed, leaving 1918 responses. The sample was further reduced to 1,395 respondents to limit the sample to caregiver reports. Of note, parents/caregivers were not restricted from participating in both sampling windows; however, only one parent/caregiver response per patient was included per sample. Demographic information for patients in the Time 2 sample can be found in Table 1.

### Questionnaire

A parent/caregiver experience questionnaire was developed by a QI committee, drawing from prior questionnaires and de novo items. The questionnaire consisted of a combination of the Likert scale (five-point; 1-strongly disagree to 5-strongly agree) and open response questions. This project focused on the responses to the following two statements: (1) “Overall, I am satisfied with the service (s) I received during the appointment.”; (2) “I would use telehealth services again, even if an in-person appointment was an option.”

### Procedure

Questionnaire invitations were emailed to parents/caregivers *via* Qualtrics Survey Platform (17) and included appointment-specific details extracted from the hospital's medical record system, including the provider's name, department/clinic, and date. For patients with multiple appointments in a survey window, the most recent visit was prioritized to optimize respondent recall and ensure proportional representation of clinical visit types across larger and smaller clinics.

### Demographic Predictors

#### Race

Of the 23 racial/ethnic categorical choices recorded in the electronic medical record, racial designations were condensed for the purposes of analysis: *White, Black, Multi-racial, Unknown*, and *Other*. Those who endorsed more than one race were classified as *Multi-racial*. Those for whom race data were unavailable were classified as *Unknown*. *Other* includes those who endorsed only one race other than white or black.

#### Age

Patient age was based on the difference between the date of birth and age at appointment. Patient age groups used in the supplemental analysis were as follows: ages 0–5, 6–10, 11–15, and 16–21.

#### Service Type

Appointments occurred in 83 different clinics throughout the hospital system, among four larger service type categories. Behavioral health services were split into two service categories. *Behavioral Health: General* includes behavioral health services (e.g., family therapy, behavioral consultation, counseling, etc.,) for children with common behavioral diagnoses, including attention-deficit/hyperactivity disorder, depression, anxiety, conduct disorder, adjustment disorder, etc. *Behavioral Health: Developmental* includes more specialized behavioral health services (e.g., applied behavior analysis) for children with developmental disorders such as intellectual disability, feeding disorders, and an autism spectrum disorder. The third group, *Medicine*, was comprised of visits primarily provided by physicians and nurses (including psychiatrists and nurse practitioners). Finally, the fourth group, *Therapy Services*, included occupational therapy (OT), speech therapy, physical therapy (PT), audiology, and nutrition.

#### Insurance Type

Insurance payor groups were categorized into a *Commercial* insurance group, a *Public* insurance group (i.e., Medicaid, Managed Medicaid, and Medicare), and *Military* insurance (i.e., a Department of Defense plan/TRICARE). Of note, patients in the military group had the most previous experience with telemedicine-based services, as TRICARE covered telemedicine in patients' homes prior to the COVID-19 pandemic.

#### Proximity to Hospital

Patient proximity to the hospital was quantified using three proximity zones. The boundaries encompassing the city proper were identified as zone 1 (i.e., *Close Proximity*). The counties immediately surrounding/adjacent to the city were identified as zone 2 (i.e., *Adjacent/Surrounding*). Finally, zone 3 (*Distal*) was identified as those counties (both in- and out-of-state) extending beyond zones 1 and 2. The Distal zone 3 included individuals from states adjacent to the hospitals as well as a wider national distribution.

### Statistical Analysis Plan

This study made use of a convenience sample, comprised solely of caregivers who responded to the two target items noted in the *Questionnaire* section above. Data were initially reviewed in Microsoft Excel (18) and further analyzed in IBM SPSS Statistics version 27.0 (19). Parametric and nonparametric tests were used to test for any demographic differences between the two samples. Analysis of Covariance (ANCOVA) was then used to determine if parent responses to two key survey items, measuring service satisfaction and willingness to use telemedicine (“telehealth” in survey items) services again, differed based upon patient demographic characteristics. The age of the child was used as a covariate in the ANCOVA model. Bonferroni-corrected *post-hoc* pairwise comparisons further described between-group differences. Additional supplemental ANOVA was used to examine response differences among parents with children in discrete age categories (i.e., ages 0–5, 6–10, 11–15, and 16–21). For reference, partial eta squared ($\eta_{p}^{2}$) was used to determine effect sizes: small (0.01), medium (0.06), and large (0.14).

## Results

### Time 1, Early Pandemic Sample

The *Time 1* sample had a mean age of 10.12 years old (SD = 4.84) and two-thirds (67.1%) of the sample were male. Among insurance groups, the proportion of patients within race groups differed [*X*^2^(8) = 179.7, *p* < 0.001] due to an overrepresentation of white patients in the commercial insurance group and an overrepresentation of black patients in the public insurance group. Mean patient age differed across service types [(*F* (3, 1307) = 76.3, *p* < 0.001], with significantly younger children in the therapy services group (M age = 6.4 years) compared to all other groups, and significantly younger children in the behavioral health: developmental (M age = 9.1 years) group compared to the behavioral health: general (M age = 11.1 years) and medicine (M age 11.4 years) groups. See Table 1 for sample details.

At *Time 1*, caregiver ratings resulted in an overall average satisfaction rating of 4.61 out of 5, and 95.5% of caregivers “agreed” or “strongly agreed” that they were satisfied with the telemedicine services they received. When controlling for age, ANCOVA revealed a very small, yet significant, main effect for insurance type (*p* = 0.032; $\eta_{p}^{2}$= 0.005), but there were no main effects found for age, race, service type, insurance type, or patient proximity to the hospital. A *post-hoc* comparison revealed that caregivers in the military insurance group provided somewhat stronger (*p* < 0.05) satisfaction ratings (4.79, SD = 0.45) compared to the commercial (4.61, SD = 0.73) and public (4.56, SD = 0.69) insurance groups.

**Figure 1:**
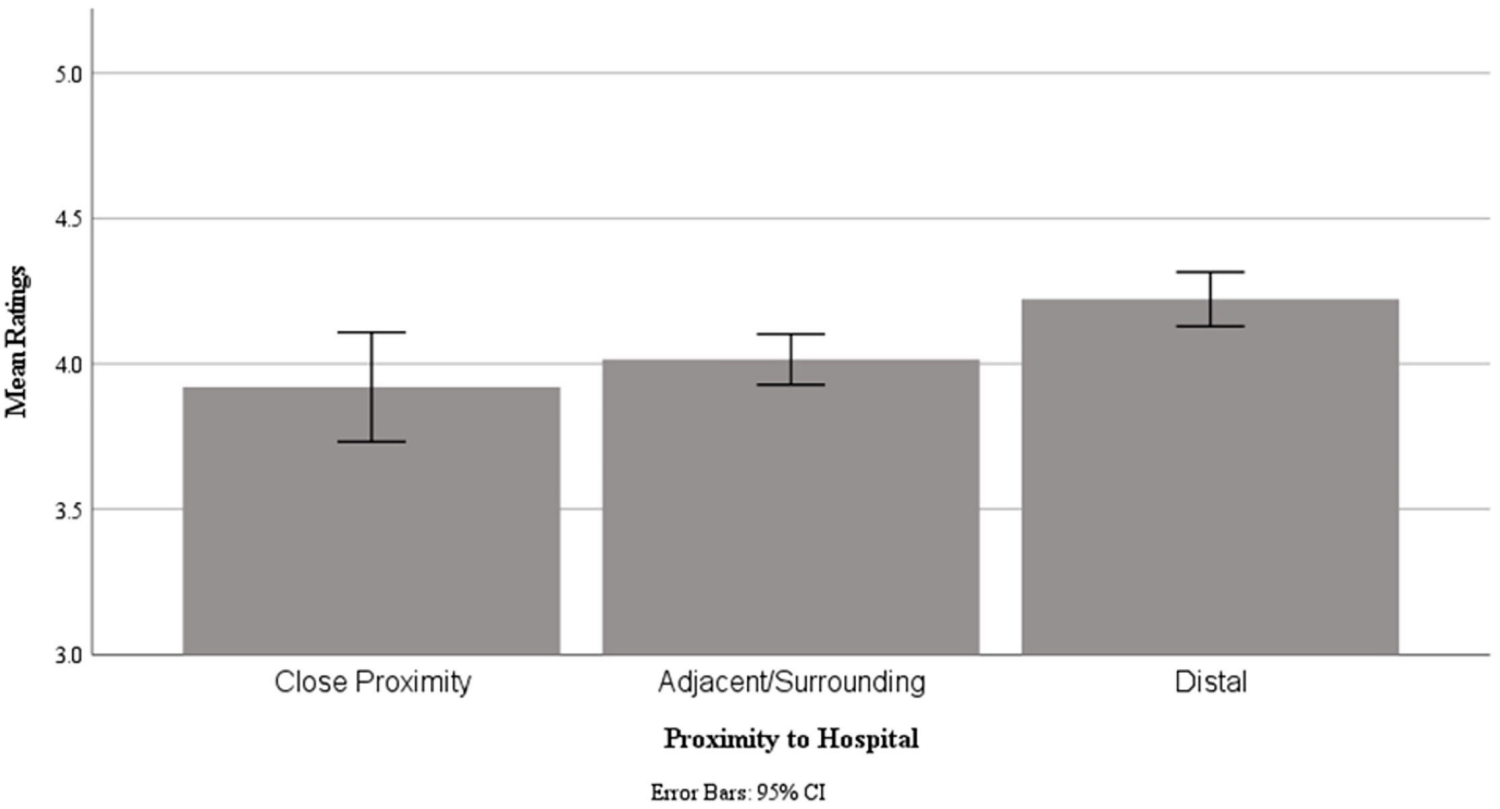
COVID-19 Time 2 (Winter Surge) Sample, Mean caregivers ratings for item “I would use telemedicine services again, even if an in-person appointment was an option” by proximity to hospital.

When asked if they would use telehealth again (even if an in-person appointment were an option), three quarters (77.1%) of caregivers agreed or strongly agreed that they would use telehealth services over a future in-person appointment, 12.5% were neutral, and a small number disagreed (7.1%) or strongly disagreed (3.3%). The ANCOVA (Table 2) revealed very small but significant effects for the insurance group (*p* = 0.029; $\eta_{p}^{2}$ = 0.005), and type of service (*p* = 0.017; $\eta_{p}^{2}$ = 0.008). There was no significant main effect of race or patient proximity to the hospital. Age was significant in the model and had a small effect size (*p* < 0.001; $\eta_{p}^{2}$ = 0.011). *Post-hoc* comparison revealed lower ratings (all *p* < 0.001) for the therapy service group compared to both behavioral health groups (see Figure 1). In terms of insurance, parents of patients with commercial insurance (4.12, SD = 1.16) provided lower (*p* = *0*.031) ratings than those with public insurance (4.26, SD = 0.97).

**Table 2:** COVID-19 Time 1 (Early Pandemic) Sample: ANCOVA (controlling for age) involving item “I would use telemedicine services again, even if an in-person appointment was an option.”

**Source**	**df**	***F***	**Sig**.	** $\eta_{\text{p}}^{2}$ **
Corrected Model	12	4.272	.000	.038
Intercept	1	1,944.071	.000	.600
Service Type	3	3.400	.017	.008
Patient Proximity to Hospital	2	2.595	.075	.004
Race	4	1.229	.297	.004
Insurance Type	2	3.547	.029	.005
Age	1	13.962	.000	.011
Error	1,298			
Total	1,311			
Corrected Total	1,310			

*df, degrees of freedom, Sig = significance; $\eta_{\text{p}}^{2}$ = partial eta squared; ^a^R^2^ = 0.038 (Adjusted R Squared = 0.029). For reference, $\eta_{\text{p}}^{2}$ effects sizes are small (0.01), medium (0.06), and large (0.14)*.

### Time 2, Winter Surge Sample

As shown in Table 1, children in the Time 2 sample had a mean age of 9.75 years (SD = 4.83) and were 60.6% male. As was the case at Time 1, race at Time 2 differed among insurance types [*X*^2^ (8) = 129.2, *p* < 0.001], due to an overrepresentation of white patients in the commercial insurance group and an overrepresentation of black patients in the public insurance group. Age differed among service types [F (3, 1391) = 76.6, *p* < 0.001], with pairwise comparison revealing significantly younger children in the therapy services group (M age = 5.2 years) compared to all other groups, and in the behavioral health: developmental (M age = 8.0 years) group compared to the behavioral health: general (M age = 10.9 years) and Medicine (M age = 10.5 years) groups.

At Time 2, 94.2% of caregivers “agreed” or “strongly agreed” that they were satisfied with the telemedicine services they received. ANCOVA revealed a very small effect for the race (*p* = 0.030; $\eta_{p}^{2}$ = 0.008), but not for the service type, insurance type, or patient proximity to the hospital. The covariate age was significant in the model and had a very small effect size (*p* < 0.05; $\eta_{p}^{2}$ = 0.003). Comparison of the race groups did not survive *post-hoc* Bonferroni-correction.

When asked if they would use telehealth again, 74.6% of caregivers “agreed” or “strongly agreed”, 13.0% were neutral on this item, and a considerable number “disagreed” (8.1%) or “strongly disagreed” (4.3%). When controlling for age, ANCOVA (Table 3) revealed small but significant effects for patient proximity to the hospital (*p* = 0.004; $\eta_{p}^{2}$ = 0.008) and type of service (*p* = 0.002; $\eta_{p}^{2}$ = 0.011), but not for insurance type or race. Age was a significant covariate in the model, with a small to medium effect size (*p* < 0.001; $\eta_{p}^{2}$ = 0.026). *Post-hoc* Bonferroni-corrected pairwise comparison revealed significantly (*p* = 0.004) higher ratings from caregivers in proximity zone 3 (distal counties or states) compared to zone 2 (surrounding or adjacent counties; **Figure 3**). *Post-hoc* comparison did not reveal significant differences between service types.

**Table 3:** COVID-19 Time 2 (Winter Surge) Sample: ANCOVA (controlling for age) involving item “I would use telemedicine services again, even if an in-person appointment was an option.”

**Source**	**df**	***F***	**Sig**.	**$\eta_{\text{p}}^{2}$d**
Corrected model	14	9.09	.000	.073
Intercept	1	2012.59	.000	.593
Service type	3	5.09	.002	.011
Patient proximity to hospital	2	5.49	.004	.008
Race	4	2.18	.069	.006
Insurance type	2	2.93	.054	.004
Age	1	36.88	.000	.026
Error	1,380			
Total	1,393			
Corrected total	1,392			

### Supplemental Analysis

As described earlier, significant main effects were noted at Time 1 and Time 2 for patient age and service type when parents were asked if they would use telemedicine again in the future. Supplemental two-way ANOVA analyses were run using these two independent variables to further investigate these variables and their potential interactions. For each analysis, the age of the patient was categorized as follows: age 0–5, 6–10, 11–15, and 16–21.

**Figure 2:**
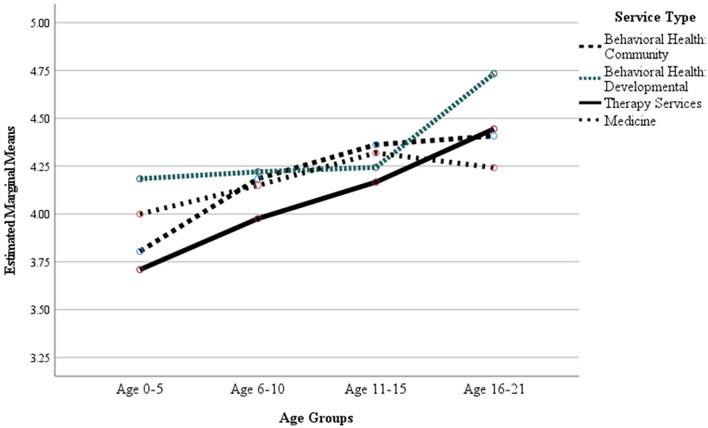
COVID-19 Time 1 (Early Pandemic) Sample, Mean caregiver ratings for item “I would use telemedicine services again, even if an in-person appointment was an option” by age group and service type.

**Figure 3:**
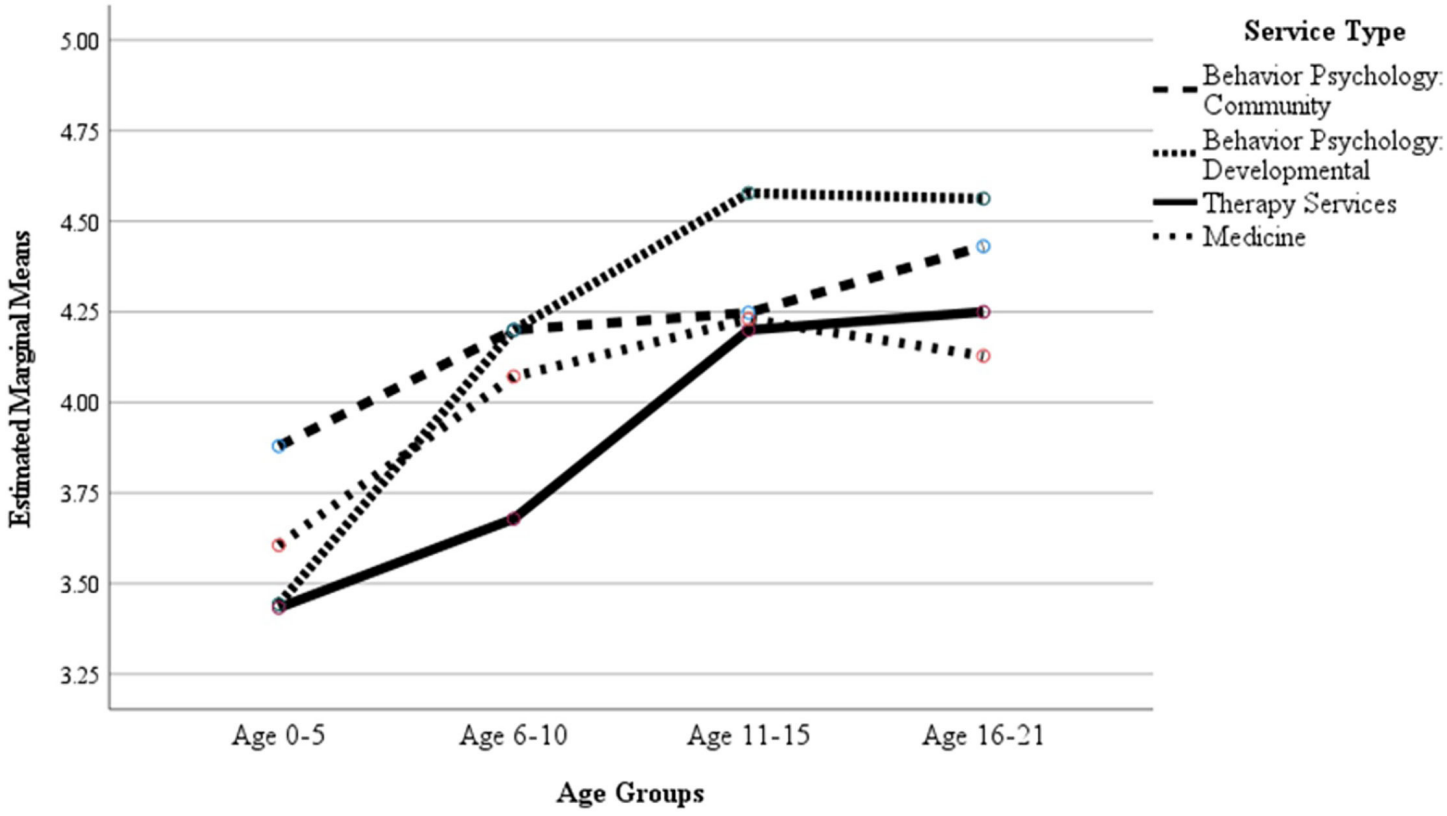
COVID-19 Time 2 (Winter Surge) Sample, Mean caregivers ratings for item “I would use telemedicine services again, even if an in-person appointment was an option” by age group and service type.

At Time 1 (Figure 2), the age group of the patient was significant (*p*<0.001; $\eta_{p}^{2}$ = 0.014) but not the service type (*p*=0.310) or the age group by service type interaction (*p* = 0.751). At Time 2 (Figure 3), both patient age group (*p* < 0.001; $\eta_{\text{p}}^{2}$ = 0.039) and service type (*p* = 0.034, $\eta_{\text{p}}^{2}$ = 0.009) were significant, but not the age group by service type interaction (*p* = 0.177).

## Discussion

The rapid upscaling of telemedicine-based services has created an unparalleled opportunity to evaluate the use of outpatient, hospital-based telemedicine services across a wide range of patient groups and hospital services. As caregiver attitudes about telemedicine were expected to evolve over the course of the COVID-19 pandemic, this quality improvement study used a repeated cross-sectional design and captured patient experience data at the beginning of the pandemic during the initial scaling-up of telemedicine care delivery (Time 1, Early Pandemic), and several months later in the pandemic when both patients and providers had more experience with telemedicine technology (Time 2, Winter Surge).

As anticipated, caregivers of children seen for outpatient telemedicine care reported very high levels of overall satisfaction both in the initial months of the COVID-19 pandemic (95%) and 6–9 months after its onset (94%). Of note, patient satisfaction ratings at the beginning of the pandemic were marginally higher among the caregivers of patients with military insurance compared to those with commercial and public insurance. This small effect is potentially attributable to pre-pandemic, reimbursable exposure of military-insurance families to telemedicine and their subsequently more experienced providers. It is worth noting that the difference in satisfaction ratings among insurance groups was no longer evident during the Time 2 winter surge, suggesting that increased caregiver/patient exposure to and/or provider experience with caregiver telemedicine contributed to the ensuing parity in satisfaction ratings between insurance groups.

There were high levels of overall satisfaction with telemedicine-based visits expressed by caregivers at both time points in this study, suggesting consistent satisfaction with telemedicine throughout the COVID-19 pandemic's first 9 months in the U.S. While there was an impressively large proportion of caregivers (77.1 and 74.6%, respectively) who agreed that they would likely use telemedicine again (even if on-site appointments were an option), there was more response variability on this item. Of note, caregivers of children between the ages of 0 and 5 provided the lowest ratings of willingness to use telemedicine again, with small and medium effect sizes noted during Time 1 and Time 2, respectively. Anecdotal reports from telemedicine-hesitant caregivers of young children suggest several areas of concern, including difficulty getting their child to pay attention and maintaining engagement with the provider. These reports are consistent with a recent survey of 271 global pediatric clinicians, in which 56.5% noted distractions at home as a barrier to the use of telemedicine services during the COVID-19 pandemic (20). Combined with similar conclusions by Tenforde et al. (9), these potential hinderances justify further exploration into the delivery of telemedicine to younger children and their families.

The present study revealed that some hospital service types (at least in their current delivery models) might be a better fit for telemedicine compared to others. Indeed, when examined in isolation, caregiver ratings of willingness to use telemedicine again were considerably lower when the patient was seen for services typically considered “hands on” (e.g., OT, PT, and speech therapy). Conversely, ratings were higher from caregivers whose children received verbally based, behavioral health services. These findings, however, are complicated by pre-existing differences in patient ages among service type groups, in that there were significantly younger children in the therapy services group compared to all other groups. At both timepoints, caregivers of older children were more enthusiastic about using telemedicine again compared to caregivers of younger children. Even after controlling for patient age, the statistical model revealed a small effect size of service type upon the willingness to use telemedicine again. Taken together, both age and therapy type appear to play an independent role in contributing to parent/caregiver satisfaction with telemedicine services and willingness to use them again.

These findings have implications for hospital efforts moving forward. Innovations are needed to adapt different types of “hands-on” therapy services for telemedicine, particularly for pre-school and early school-aged children. In the meantime, these data may signal the need to prioritize younger children for onsite/in-person care where possible, particularly for those patients receiving OT, PT, and speech therapy services.

This study has several notable strengths. Of note, this study explored the use of and satisfaction with telemedicine services across an entire hospital system, capturing data from multiple disciplines and a wide patient age span. This allows for the comparison of parent/caregiver telemedicine satisfaction between disciplines and age groups, which assists in the identification of relative strengths and weaknesses within the telemedicine service modality. Additionally, this study made use of a repeated, cross-sectional design, which allowed for the examination of satisfaction with telemedicine services at different time points, as well as our hospital's provision of telemedicine services, during the COVID-19 pandemic. Lastly, this study had a notable sample size, thus providing more reliable insight into parent/caregiver responses.

This study is not without its limitations. The foremost limitation is the potential for selection bias due to the low and inconsistent response rate across timepoints (24.9% for Time 1, and 11.9% for Time 2) and sampling from a single hospital site. Additionally, satisfaction ratings were only received from caregivers who agreed to a telemedicine visit, and not those who declined telemedicine services, preventing us from identifying other possible barriers to accessing care. Over and above the demographic variables included in this study, there are other variables that could account for variability in caregiver ratings of telemedicine, including language and socioeconomic inequities in access to telemedicine care (21–23). There may also be provider-related variables that impacted the quality of experience for patients and/or caregivers, thus influencing willingness to use telemedicine again (6). Since pediatric self-reported ratings of experience have been found to differ from those of their caregivers (24, 25) and have been found to be feasible to collect (26), future projects should consider surveying pediatric patients in addition to caregivers. Lastly, future studies should further examine caregiver telemedicine satisfaction among patients with complex or chronic conditions, as these families may differentially value telemedicine services given the number of appointments and/or related travel inherent to the management of these conditions.

In summary, this study provides overwhelmingly positive feedback from parents/caregivers indicating satisfaction with the quality of telemedicine services received and an interest in utilizing telemedicine services in the future, even when on-site appointments are available. The current study identified several areas deserving of attention and innovative effort to adapt the conduciveness of certain therapy services to telemedicine, particularly for younger children. It is clear that telemedicine is becoming a more permanent modality of care, but as in-person appointments become safer to resume, it is critical that patient and caregiver telemedicine experiences must continue to be researched in order to best inform efforts to expand accessibility to all families, regardless of patient age or service type.

## Data Availability Statement

The datasets presented in this article are not readily available because the responses are directly linked to patient protected health information. Requests to access the datasets should be directed to TZ, zabela@kennedykrieger.org.

## Ethics Statement

The studies involving human participants were reviewed and approved by Johns Hopkins Institutional Review Boards. Written informed consent for participation was not required for this study in accordance with the national legislation and the institutional requirements.

## Author Contributions

EJ, TZ, LJ, JK, CS, JS, JT, and ED contributed to the conception and design of the study. EJ managed the collection and storage of data. LK, TZ, JT, and LJ performed the statistical analyses. EJ and TZ wrote the first draft of the manuscript. JC, JS, RP, JT, and CS wrote sections of the manuscript. All authors contributed to manuscript revision, read, and approved the submission version.

## Funding

This study was conducted with the support of the Orokawa Foundation and the Isadore and Bertha Gudelsky Family Foundation. This work was also partially supported by the Intellectual and Developmental Disabilities Research Center at Kennedy Krieger Institute and Johns Hopkins University (P50 HD103538).

## Conflict of Interest

The authors declare that the research was conducted in the absence of any commercial or financial relationships that could be construed as a potential conflict of interest.

## Publisher's Note

All claims expressed in this article are solely those of the authors and do not necessarily represent those of their affiliated organizations, or those of the publisher, the editors and the reviewers. Any product that may be evaluated in this article, or claim that may be made by its manufacturer, is not guaranteed or endorsed by the publisher.
